# Intracholecystic Papillary Neoplasm: A Rare Case Report

**DOI:** 10.7759/cureus.69955

**Published:** 2024-09-22

**Authors:** Shubham N Bargaje, Harshal Ramteke, Aishwarya K Tare

**Affiliations:** 1 Surgery, N.K.P. Salve Institute of Medical Sciences and Research Center, Nagpur, IND

**Keywords:** cholecystectomy, gall bladder, gall bladder carcinoma, gall bladder polyp, intracholecystic papillary neoplasm

## Abstract

Intracholecystic papillary neoplasm (ICPN) is a mass-forming, noninvasive epithelial premalignant neoplasm arising from the gallbladder mucosal lining and projecting into the lumen of the gallbladder. It is potentially fatal and if left untreated can progress to invasive gallbladder carcinoma. Incidentally ICPN is found on imaging or during postoperative histological evaluation and is likely to be missed preoperatively. We present a rare case of ICPN in a 70-year-old female patient who was admitted with complaints of pain in the right hypochondrium since one month and was postoperatively diagnosed with ICPN through histopathological examination. We discuss the need for early intervention in gallbladder polypoidal masses to prevent the conversion of benign or preinvasive tumors to malignancy.

## Introduction

Adsay et al. (2012) first described intracholecystic papillary neoplasm (ICPN) [1], and later the WHO (2019) recognised it as a distinct type of preinvasive gallbladder neoplasm under the name of ICPN [2]. Intraepithelial neoplasia are preinvasive neoplastic lesions found throughout the digestive system [3]. When discovered in the gallbladder, they are referred to as ICPNs [3]. ICPN is a rare gallbladder lining tumor with better prognosis [4]. However, due to its malignant potential, the correct diagnosis and classification are crucial. ICPN is a rare disease of the gallbladder seen in <1% of all cholecystectomies [1], which more commonly involves the fundus and body of the gallbladder [5]. It is usually incidentally detected postoperatively after histopathological examination. Many cases are asymptomatic, and few present with pain in the right upper quadrant of the abdomen [6]. ICPN shows a preponderance towards females and commonly presents in older age with a mean age of presentation of around 61 years [1]. Here, we discuss a case of a 70-year-old female patient who presented with pain in the right upper quadrant of the abdomen and changes in the radiological imaging of the gallbladder.

## Case presentation

A 70-year-old female patient presented with complaints of pain in the right upper quadrant of abdomen for one month. The pain was gradual in onset and intermittent in nature. Pain was not associated with consumption of meals, and no episodes of nausea or vomiting were reported.

On admission, the haemodynamic parameters included a temperature of 98.7°F, a pulse of 90 bpm, a respiratory rate of 16 breaths per minute, and a blood pressure of 130/90 mmHg. On physical examination, the abdomen was normal with no tenderness or mass palpable in the abdomen. All haematological and biochemical investigations (complete blood count, liver function test, renal function test, lipid profile) were normal.

**Figure 1:**
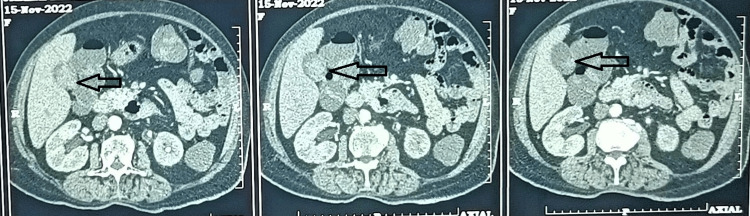
Contrast-enhanced computed tomography (CECT) (AP) showing polypoidal lesion (arrows)

USG of the abdomen and pelvis revealed an ill-defined hypoechoic lesion in the gallbladder, suggestive of a neoplastic etiology or tumefactive sludge. The contrast-enhanced computed tomography (CECT) abdomen and pelvis showed a distended gallbladder with an ill-defined heterogeneous predominantly isodense (Hounsfield unit (HU) +15 to +35) polypoidal lesion of about 3.4 x 2 cm noted in the body and neck of the gallbladder. A heterogenous enhancement of the lesion was found in a post-contrast study. Common bile duct (CBD) appears normal in caliber (Figure 1).

A patient with fitness for surgery underwent an open cholecystectomy. The procedure was well tolerated, and the resected gallbladder specimen was sent for histopathology examination.

**Figure 2:**
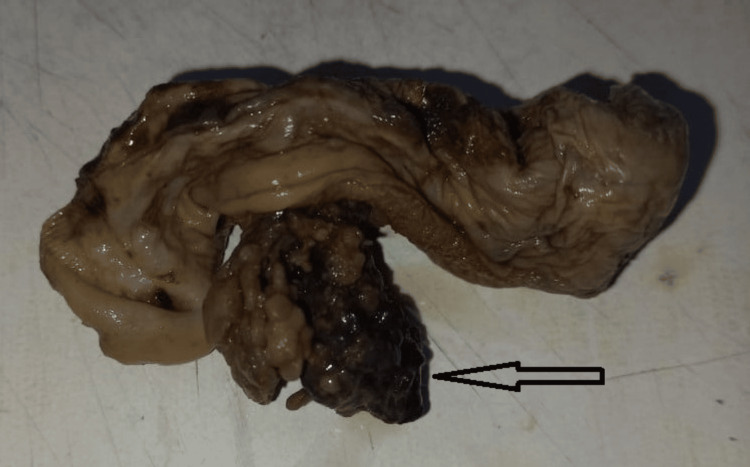
Cut section of gallbladder showing exophytic growth (arrow).

The gross pathology indicated that the gallbladder had multiple, irregular, greyish-brown friable tissue bits. A lumen shows an irregular exophytic mass with friable greyish-brown papillae attached to the mucosal lining of the gallbladder. The polyp measured about 3.4 X 2.5 X 1.0 cm (Figure 2).

**Figure 3:**
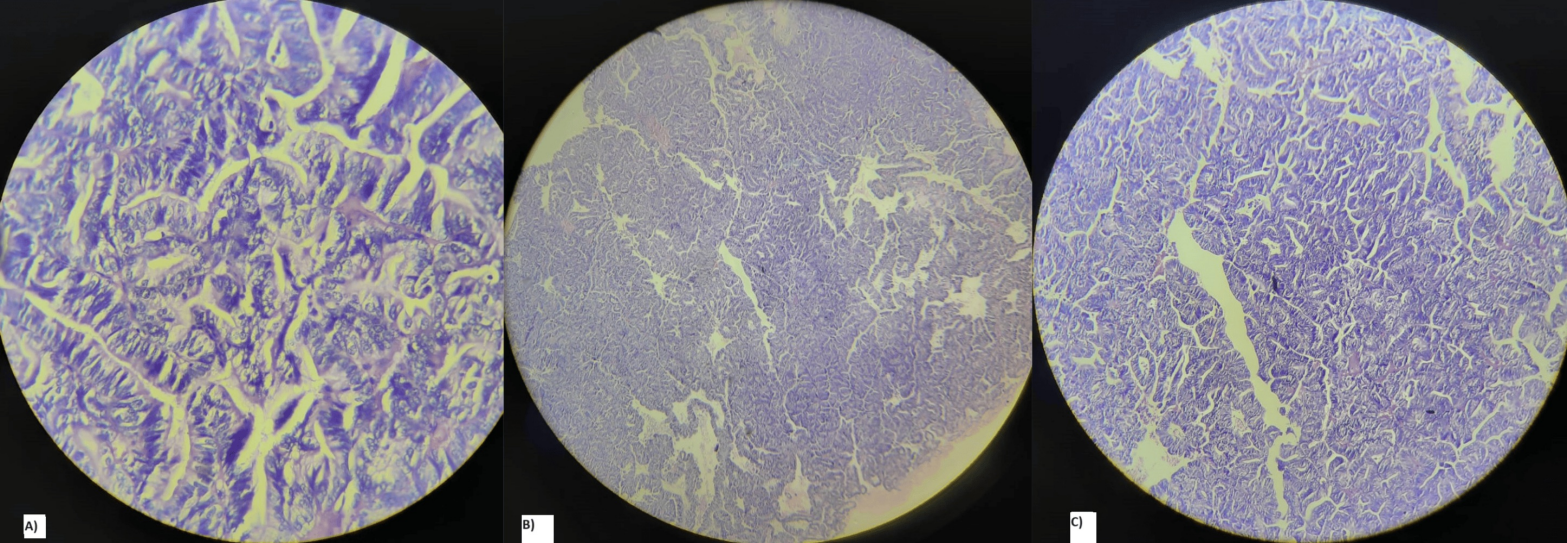
Histopathological findings: A) Tall columnar cells with oval nuclei, B) Hyperplastic mucosa, C) Polypoidal mass showing tubulo-papillary structures arranged back to back.

Microscopic examination revealed a polypoidal lesion showing tubulo-papillary structures arranged back to back and lined by pseudostratified tall columnar cells with oval nuclei with or without nucleoli and basophilic cytoplasm (Figure 3). 

Histopathological evaluation confirmed the diagnosis of Intra-cholecystic papillary neoplasm with high-grade intraepithelial neoplasia. Patient recovered uneventfully and was discharged on postoperative day nine.

## Discussion

ICPN is a rare premalignant neoplasm. Typically, it presents as intermittent right upper quadrant (RUQ) pain with a detectable intraluminal mass (≥1 cm) on conventional imaging, such as USG or contrast CT [4]. As many as 50% of ICPN cases were detected incidentally, and approximately 10% were missed in imaging studies [4]. Etiological factors have not been identified, but the few identified risk factors are advanced age and female preponderance [2].

Polypoid masses in the gallbladder, including benign, malignant, and non-neoplastic lesions, have been referred to as gallbladder polyps [7]. ICPN is a polypoid lesion in the gallbladder, but differentiating between gallbladder polyps and ICPN is difficult. The diagnostic protocol of ICPN involves a combination of in-depth clinical evaluation, imaging studies, and histopathological analysis [4,8].

A gross examination of the specimen showed friable excrescences loosely attached to the lumen, which may be mistaken for sludge or debris in the lumen [2]. The median tumor size is around 2.2 cm [1]. Microscopically it reveals three different growth patterns, papillary, tubular or tubule-papillary, and four morphological patterns described as biliary, intestinal, gastric and oncocytic [2]. It exhibits many similarities to pancreatic intraductal papillary mucosal neoplasms (IPMN), as they demonstrate a spectrum of variable configurations, different cell lineages (often in a mixture), and dysplastic changes [9]. ICPN-associated invasive carcinomas are relatively indolent neoplasms with a significantly better prognosis than conventional (pancreatobiliary type) gallbladder carcinomas [2].

In 2018, Hazarika et al. performed a five-year retrospective study on ICPN that revealed papillary configuration, biliary or foveolar phenotypes. The presence of high-grade dysplasia is considered a signiﬁcant pathologic risk factor for associated invasive carcinoma [2,10]. Although most cases are asymptomatic, ICPN is a premalignant lesion that can evolve into invasive carcinoma in about 6.4% of the cases [11]. KRAS mutation is common [12], and STK11, CTNNB1 and APC mutations have also been identified [2].

Currently, USG, CT, and MRI are the standards of imaging investigations for detecting ICPN lesions [13]. USG detects the presence of gallbladder polyps or masses and assesses their size, location, and appearance [14]. CT provides information on the extent of the tumor invasion into nearby structures and distant or lymph node metastasis [11]. The classic treatment of ICPN involves surgical resection in the form of cholecystectomy [4].

If undetected, ICPN transforms into high-grade invasive carcinoma, leading to adverse patient outcomes due to its asymptomatic nature and a lack of screening guidelines despite known risk factors [4]. At final pathology, the rate of invasive cancer amongst patients with ICPN of the gallbladder was 57% [1]. Even when ICPN with associated invasive carcinoma is only considered, its overall survival outcome is incomparably better than that of the non-ICPN associated ordinary-type gallbladder adenocarcinoma, which has a five-year survival rate ranging from 18 to 30% [15].

This case report aims to highlight the need for early screening and surgical intervention to prevent conversion to invasive carcinoma. It focuses on clinical and pathological findings and emphasizes that cholecystectomy is the only treatment modality for ICPN patients. Therefore, treatment should not be delayed. Either open or laparoscopic cholecystectomy should be performed when a polypoid lesion in the gallbladder is identified on abdominal ultrasound and confirmed by abdominal contrast-enhanced CT, as delays in surgical intervention can result in progression to invasive carcinoma.

## Conclusions

This case report presents a rare instance of ICPN, highlighting that this diagnosis is challenging to make without histopathological examination. The report contributes clinical and pathological information to the existing literature and emphasizes the need for research into risk factors, etiological factors, and treatment protocols. It also raises awareness that intra-luminal polypoidal gallbladder masses should include ICPN in the differential diagnosis and that prompt treatment with cholecystectomy is crucial, as early intervention can prevent progression to invasive carcinoma.
